# A study on the efficacy of acupuncture combined with erector spinae plane blockade in patients undergoing thoracoscopic lung lobectomy

**DOI:** 10.1097/MD.0000000000047325

**Published:** 2026-01-23

**Authors:** Jian Huang, Shibiao Chen, Liqin Wang, Weicheng Liu, Yanping Zhan, Mengying Xiong, Jin Zhang

**Affiliations:** a Department of Anesthesiology, The First Affiliated Hospital of Nanchang University, Nanchang City, Jiangxi Province, China; b Department of Preventive Treatment Center, The First Affiliated Hospital of Nanchang University, Nanchang City, Jiangxi Province, China.

**Keywords:** acupuncture anesthesia, erector spinae plane block, postoperative nausea and vomiting, postoperative pain, stress response, thoracoscopic lung resection

## Abstract

This study aims to investigate the effects of acupuncture anesthesia combined with erector spinae plane block (ESPB) on postoperative pain, nausea severity, and stress responses in patients undergoing thoracoscopic lung lobectomy, providing evidence for optimizing anesthesia protocols. Forty patients who underwent elective unilateral thoracoscopic lung lobectomy at the First Affiliated Hospital of Nanchang University (American Society of Anesthesiologists I–II) during the study period were retrospectively identified. According to the perioperative analgesic strategy recorded in the clinical database, patients were categorized into the ESPB-only group (E group) and the acupuncture combined with ESPB group (A + E group). The E group underwent ESPB at the T5 transverse process level (0.5% ropivacaine), while the A + E group received ESPB combined with electroacupuncture at bilateral Hegu, Houxi, Zhigou, and Neiguan acupoints (sparse-dense wave 2/100 Hz) until the end of surgery. The following parameters were recorded for both groups: intraoperative remifentanil dosage, visual analog scale scores at 4, 8, 12, 24, and 48 hours postoperatively, nausea numerical rating scale scores, patient-controlled analgesia button presses, sufentanil dosage, plasma neutrophil concentration before and 24 hours after surgery, and postoperative hospital stay. In the A + E group, visual analog scale scores and numerical rating scale scores at rest and during coughing at all postoperative time points were significantly lower than those in the E group (*P* < .05); The A + E group had fewer patient-controlled analgesia presses, lower postoperative sufentanil dosage, and lower intraoperative remifentanil dosage than the E group (*P* < .05); the A + E group had a lower increase in plasma neutrophil concentration at 24 hours postoperatively than the E group (*P* < .05); and the A + E group had a shorter postoperative hospital stay than the E group (*P* < .05). Acupuncture anesthesia combined with erector spinae plane block effectively reduces postoperative pain and nausea in patients undergoing thoracoscopic lung lobectomy, reduces opioid consumption, inhibits postoperative stress responses, and promotes postoperative recovery.

## 1. Introduction

Video-assisted thoracoscopic surgery (VATS) significantly reduces surgical trauma due to its minimally invasive nature. However, the incidence of moderate to severe postoperative pain remains as high as 50% or more, severely impacting patients’ early mobility, respiratory function recovery, and quality of life.^[1,2]^ Pain not only stems from incision-related mechanical stimulation but is also closely associated with thoracic drainage tube stimulation, visceral traction pain, and inflammatory responses.^[3-7]^ Currently, multimodal analgesia strategies are central to postoperative management in thoracic surgery, with regional nerve block techniques being particularly important. The erector spinae plane block (ESPB), as an emerging fascial plane block technique, involves injecting local anesthetics into the deep layer of the erector spinae muscles to block the dorsal and communicating branches of the spinal nerves, effectively covering somatic pain in the T1–T12 segments.^[8,9]^ However, ESPB alone has limited efficacy in suppressing visceral pain and cannot completely avoid opioid-related side effects (such as nausea, vomiting, and intestinal paralysis).^[10]^

Acupuncture anesthesia, a unique achievement of traditional Chinese medicine, involves mechanisms such as activating the endogenous analgesic system (e.g., promoting the release of β-endorphins and serotonin), regulating the hypothalamic-pituitary-adrenal axis, and inhibiting the expression of inflammatory factors.^[11-13]^ Clinical studies have shown that electroacupuncture stimulation can reduce opioid requirements by 30% in patients undergoing thoracoabdominal surgery, while also reducing the incidence of postoperative nausea and vomiting (PONV).^[14]^ Acupoints such as Hegu (LI4) and Neiguan (PC6) have been proven to have clear central analgesic and antiemetic effects.^[15,16]^ Therefore, combining acupuncture anesthesia with ESPB may optimize analgesic effects through a peripheral-central synergistic mechanism while reducing stress responses and opioid side effects, aligning with the principles of enhanced recovery after surgery.^[17]^ This study aims to explore the application value of acupuncture anesthesia combined with ESPB in VATS, evaluate its comprehensive effects on postoperative pain, nausea severity, and stress responses, and provide clinicians with an optimized multimodal analgesic regimen.

## 2. Materials and methods

### 2.1. Study population

This study retrospectively analyzed 40 patients who had previously undergone unilateral thoracoscopic lung lobectomy at the First Affiliated Hospital of Nanchang University between May 2024 and November 2024 were selected. The study was approved by the hospital’s ethics committee (approval number: IIT [2024] Linlunshen No. 201), and informed consent was obtained from the patients and their families.

Inclusion criteria: first-time recipients of needle anesthesia; American Society of Anesthesiologists I–II classification; age 30 to 68 years; body mass index ≤ 25 kg/m^2^; signed informed consent form.

Exclusion criteria: skin infection or damage at the puncture site/acupoint; coagulation dysfunction (international normalized ratio > 1.3); participation in other clinical trials within the past 4 weeks; communication barriers, severe ventilatory dysfunction, active respiratory tract infection, infectious diseases, severe heart, liver, or kidney dysfunction, uncontrolled diabetes, allergy to local anesthetics, or history of mental illness.

The sample size of 40 patients represented all eligible cases within the study period who met the predefined inclusion and exclusion criteria. No additional case selection or matching was performed, consistent with the retrospective observational design.

### 2.2. Grouping and methods

This study adopted a retrospective observational design. A total of 40 eligible patients who underwent thoracic-level analgesic management within the study period were included and categorized into 2 groups based on the analgesic modality recorded in the medical electronic database: the E group (n = 20), who received ultrasound-guided ESPB alone, and the A + E group (n = 20), who received acupuncture combined with ESPB. In the E group, ESPB was performed at the T5 transverse process level with 0.5% ropivacaine (20 mL), followed by general anesthesia after approximately 20 minutes. In the A + E group, ESPB intervention was performed following the same procedure as the E group, after which acupuncture therapy was applied immediately following successful ESPB. Bilateral Hegu, Houxi, Zhigou, and Neiguan acupoints (in accordance with GB12346-90) were selected and connected to the huatuo Brand Electronic Acupuncture Treatment Instrument (model: SDZ-II). The sparse-dense wave mode was adopted, with parameters set at 2/100 Hz, and the intensity was adjusted to the maximum pain-free level tolerable by the patient. Continuous stimulation was maintained until the end of the procedure. General anesthesia was administered 30 minutes after electroacupuncture.

Anesthesia methods – Preoperative preparation: all patients are prohibited from smoking for 2 weeks, and are prohibited from drinking for 2 hours and eating for 8 hours prior to surgery. Preanesthetic monitoring: establish peripheral venous access, administer Ringer’s solution at 6 to 8 mL/kg, and monitor peripheral capillary oxygen saturation, 5-lead electrocardiogram, end-tidal carbon dioxide, and radial artery invasive pressure. Erector spinae plane block (ESP, common to both groups): use an S-NERVE ultrasound machine (Sonosite, FUJIFILM Sonosite, Inc., Bothell, WA, USA) with a high-frequency linear probe positioned longitudinally in the sagittal plane 3 cm lateral to the T4 spinous process, targeting the T5 transverse process; A B. Braun A-type nerve block needle (B. Braun Medical AG, Melsungen, Germany) was used to advance the needle into the plane at the T5 transverse process surface and the erector spinae muscle plane using the intraplanar technique. 0.5% ropivacaine (5 mL: 0.1 g, Zhengzhou Zhaofeng Pharmaceutical Co., Ltd., Zhengzhou, China.) was injected. After confirming the spread of the local anesthetic under ultrasound guidance, general anesthesia was administered within 20 minutes. Acupuncture intervention (A + E group): After successful ESP, the bilateral Hegu, Houxi, Zhigou, and Neiguan acupoints were selected according to the national standard “Acupoint Locations” (GB12346-90); connect the huatuo Brand Electronic Acupuncture Therapy Instrument (Suzhou Medical Supplies Factory Co., Ltd., Suzhou, China), with electroacupuncture parameters: frequency AM 2/100 Hz sparse-dense wave (symmetric bidirectional pulse), stimulation intensity set to the maximum tolerable threshold in a conscious state, and continue until the end of the procedure. General anesthesia is administered 30 minutes after acupuncture. General anesthesia induction and maintenance: Induction: dexmedetomidine 0.5 μg/kg (2 mL: 0.2 mg, Jiangsu Yangtze River Pharmaceutical Group Co., Ltd., Jiangsu, China), propofol 2 mg/kg (20 mL: 200 mg, Guangdong Jiabo Pharmaceutical Co., Ltd., Guangdong, China), rocuronium bromide 0.6 mg/kg (10 mL:50 mg, Zhejiang Xianju Pharmaceutical Co., Ltd., Zhejiang, China), and sufentanil 0.3 μg/kg (1 mL:50 μg, Yichang Renfu Pharmaceutical Co. Ltd., Yichang, Hubei). After 2 minutes of apnea, a double-lumen tube was orally inserted, and intermittent positive pressure ventilation was performed using a Plus-type anesthesia machine (German Dräger Company), with a tidal volume of 6 to 8 mL/kg, a respiratory rate of 12 to 20 breaths/min, and maintenance of PETCO₂ at 35 to 45 mm Hg. Maintenance: Continuous infusion of propofol at 3 to 5 mg/(kg h) and atracurium besylate at 0.1 mg/(kg h; 10 mg, Jiangsu Hengrui Medicine Co., Ltd., Jiangsu, China). and remifentanil (1 mg, Yichang Renfu Pharmaceutical Co., Ltd., Yichang, China) at an initial concentration of 0.1 μg/(kg min), with dosage adjusted based on the PRST score (heart rate, blood pressure, sweating, and tearing) in increments of 0.05 μg/(kg min) to maintain a bispectral index of 40 to 60. Postoperative analgesia – patient-controlled analgesia (PCA) formula: sufentanil 3 μg/kg + saline diluted to 100 mL, background dose 2 mL/h, self-administered single dose 1 ml, lockout time 15 minutes.

### 2.3. Observation indicators

Surgical duration; Pain: assessed at 4, 8, 12, 24, and 48 hours postoperatively using the visual analog scale (VAS, 0–10 points; at rest and during coughing); Nausea: assessed at the same time points using the numerical rating scale (NRS, 0–10 points). Analgesia-related indicators – Postoperatively: number of effective PCA button presses within 48 hours, total consumption of sufentanil; Intraoperatively: total dose of remifentanil; Stress response indicators: plasma neutrophil concentration preoperatively and 24 hours postoperatively; rehabilitation indicators: postoperative hospital stay duration.

### 2.4. Statistical analysis

SPSS 25.0 software was used. Quantitative data were expressed as mean ± standard deviation (*x* ± *s*), and intergroup comparisons were performed using independent samples *t*-tests; repeated measures data were analyzed using repeated measures analysis of variance. Categorical data were expressed as counts (%), and chi-square tests were used. *P* < .05 was considered statistically significant.

## 3. Results

### 3.1. General information and surgical conditions

There were no statistically significant differences in age, gender, body mass index, American Society of Anesthesiologists classification, or duration of surgery between the 2 groups (*P* > .05), indicating comparability (Table 1).

**Table 1:** Comparison of general information and surgical conditions between the 2 groups.

Item	A + E group	E group	*t*/χ^2^ value	*P* value
Age (yr)	55.8 ± 8.7	57.2 ± 9.1	0.512	.612
Gender (male/female)	12/8	14/6	0.476	.490
BMI	22.3 ± 1.8	22.7 ± 1.6	0.782	.439
ASA (I/II)	9/11	8/12	0.114	.736
Duration of surgery (min)	142.5 ± 25.3	148.2 ± 28.7	0.684	.498

ASA = American Society of Anesthesiologists, BMI = body mass index.

### 3.2. Postoperative pain scores (VAS)

Resting VAS: Scores in the A + E group were significantly lower than those in the E group at 4, 8, 12, 24, and 48 hours postoperatively (*P* < .05; Table 2).

**Table 2:** Comparison of postoperative resting VAS scores between the 2 groups (points, *x̄* ± *s*).

Time point (h)	A + E group (n = 20)	E group (n = 20)	*t* value	*P* value
4	2.1 ± 0.7*	3.8 ± 1.1	6.132	<.001
8	1.9 ± 0.6*	3.5 ± 0.9	6.874	<.001
12	1.7 ± 0.5*	3.2 ± 0.8	7.211	<.001
24	1.5 ± 0.4*	2.9 ± 0.7	7.893	<.001
48	1.2 ± 0.3*	2.3 ± 0.6	7.024	<.001

VAS = visual analog scale.

* indicates a significant difference between groups (*P* < .05).

Coughing VAS: Scores in the A + E group were also significantly lower than those in the E group at all evaluation time points (*P* < .05; Table 3).

**Table 3:** Comparison of postoperative coughing VAS scores between the 2 groups (points, *x̄* ± *s*).

Time point (h)	A + E group (n = 20)	E group (n = 20)	*t* value	*P* value
4	3.2 ± 0.8*	5.1 ± 1.2	6.032	<.001
8	2.9 ± 0.7*	4.7 ± 1.1	6.782	<.001
12	2.6 ± 0.6*	4.3 ± 1.0	6.954	<.001
24	2.3 ± 0.5*	3.9 ± 0.9	7.112	<.001
48	1.8 ± 0.4*	3.2 ± 0.7	7.846	<.001

VAS = visual analog scale.

* indicates a significant difference between groups (*P* < .05).

### 3.3. Postoperative nausea scores (NRS)

Nausea NRS scores in the A + E group were significantly lower than those in the E group at 4, 8, 12, and 24 hours postoperatively (*P* < .05), but there was no statistically significant difference between the 2 groups at 48 hours (*P* > .05; Table 4).

**Table 4:** Comparison of postoperative nausea NRS scores between the 2 groups (points, *x̄* ± *s*).

Time point (h)	A + E group (n = 20)	E group (n = 20)	*t* value	*P* value
4	1.5 ± 0.6*	3.2 ± 1.0	6.782	<.001
8	1.3 ± 0.5*	2.9 ± 0.8	7.214	<.001
12	1.2 ± 0.4*	2.6 ± 0.7	7.652	<.001
24	0.9 ± 0.3*	2.1 ± 0.6	8.032	<.001
48	0.6 ± 0.2	0.8 ± 0.3	1.214	.232

NRS = numerical rating scale.

* indicates a significant difference between groups (*P* < .05).

### 3.4. Dosages of anesthetic and analgesic drugs

The intraoperative dosage of remifentanil in the A + E group was significantly lower than that in the E group (*P* < .05); the number of PCA presses within 48 hours postoperatively in the A + E group was significantly lower than that in the E group (*P* < .05); the dosage of sufentanil within 48 hours postoperatively in the A + E group was significantly lower than that in the E group (*P* < .05; Table 5).

**Table 5:** Comparison of dosages of anesthetic and analgesic drugs between the 2 groups (*x̄* ± *s*).

Item	A + E group	E group	*t* value	*P* value
Intraoperative remifentanil dosage (μg)	785.6 ± 132.4	1023.8 ± 158.7	5.213	<.001
Number of PCA presses within 48 h postoperatively (times)	12.4 ± 3.8	18.7 ± 4.5	4.782	<.001
Sufentanil dosage within 48 h postoperatively (μg)	48.3 ± 10.2	65.8 ± 12.7	4.894	<.001

PCA = patient-controlled analgesia.

### 3.5. Stress response indicators

There was no significant difference in preoperative neutrophil concentration between the 2 groups (*P* > .05). At 24 hours postoperatively, neutrophil concentrations in both groups were increased compared with preoperative levels (*P* < .05), but the increase amplitude in the A + E group was significantly lower than that in the E group (*P* < .05; Table 6).

**Table 6:** Comparison of plasma neutrophil concentrations between the 2 groups (*x̄* ± *s*, ×10**⁹**/L).

Time point	A + E group	E group	*t* value	*P* value
Preoperatively	4.1 ± 1.2	4.3 ± 1.3	0.532	.598
24 h postoperatively	8.7 ± 1.8*^,^**	10.5 ± 2.1*	3.047	.004

* indicates a significant difference compared with the preoperative level (*P* < .05).

** indicates a significant difference between groups (*P* < .05).

### 3.6. Postoperative hospital stay

The postoperative hospital stay in the A + E group was significantly shorter than that in the E group (*P* < .05; Table 7).

**Table 7:** Comparison of postoperative hospital stay between the 2 groups (d, *x̄* ± *s*).

Group	Number of cases	Postoperative hospital stay	*t* value	*P* value
A + E group	20	5.2 ± 0.8*	3.892	.001
E group	20	6.7 ± 1.1		

* indicates a significant difference between groups (*P* < .05).

## 4. Discussion

This study confirmed that the use of acupuncture anesthesia combined with ESPB (A + E) during thoracoscopic lobectomy significantly improves perioperative management compared to ESPB alone (E): Patients in the A + E group demonstrated superior postoperative analgesia, with VAS scores at all assessment time points (at rest and during coughing) within 48 hours postoperatively being significantly lower than those in the E group, This is attributed to acupuncture stimulating specific acupoints such as Hegu, Houxi, Zhigou, and Neiguan, activating the endogenous analgesic system (e.g., releasing endorphins and enkephalins), inhibiting nociceptive signal transmission, and synergizing with the regional block of ESPB to cover a broader pain pathway; Additionally, the A + E group exhibited significantly reduced nausea NRS scores in the early postoperative period (4–24 hours), as acupuncture regulates gastrointestinal function, inhibits vagus nerve excitation, and suppresses 5-HT3 release. This mechanism for preventing and treating PONV compensates for the limitations of sole nerve block and opioid medications in this regard; Furthermore, the A + E group had significantly reduced intraoperative remifentanil dosage, postoperative PCA button presses, and sufentanil consumption. The analgesic effects of acupuncture reduced the body’s response to nociceptive stimuli, thereby decreasing the need for intraoperative opioid maintenance and postoperative rescue analgesia, helping to avoid opioid-related side effects (such as respiratory depression, intestinal obstruction, and addiction risk); In terms of suppressing surgical stress responses, the increase in plasma neutrophil concentration within 24 hours postoperatively – a sensitive indicator of surgical trauma stress – was significantly lower in the A + E group compared to the E group, indicating that the acupuncture combined blockage protocol more effectively reduces systemic inflammation and stress responses caused by surgery, thereby creating conditions for the restoration of postoperative immune homeostasis; All these advantages ultimately translate into significant clinical benefits – patients in the A + E group had significantly shorter postoperative hospital stays, with good pain control, fewer episodes of nausea and vomiting, reduced opioid-related side effects, and alleviated stress responses, all of which promoted early ambulation, resumption of oral intake, and overall functional recovery. Patients in the A + E group experienced lighter postoperative pain, possibly due to: ESP blocking peripheral pain transmission of thoracic nerves, acupuncture inhibiting spinal dorsal horn neuronal activity through the “gate control” mechanism and promoting the release of endogenous analgesic substances such as endorphins.^[9-11]^ In this study, electroacupuncture used a 2/100 Hz sparse-dense waveform, combining the synergistic effects of low-frequency (promoting endorphin synthesis) and high-frequency (inhibiting pain fiber conduction). Acupuncture can suppress the vomiting center by regulating vagus nerve tension, while reducing the use of opioid medications (such as sufentanil and remifentanil), thereby indirectly lowering the incidence of nausea. In this study, the opioid dosage in the A + E group was significantly reduced, consistent with the decrease in nausea scores, validating the synergistic effect of “multimodal antiemesis.”^[12,13]^ Neutrophils are a core indicator of perioperative stress, and their elevation reflects the degree of neuroendocrine-immune activation triggered by trauma. The A + E group exhibited a slower increase in neutrophil levels, suggesting that acupuncture combined with ESP may modulate the hypothalamic-pituitary-adrenal axis to inhibit perioperative stress responses. The combination of acupuncture anesthesia and ESPB (A + E) optimized perioperative management for thoracoscopic lobectomy through a multi-target synergistic mechanism: In terms of analgesia, ESPB blocks the dorsal branches of the thoracic nerves (T1–T12) to inhibit peripheral pain input,^[14]^ while acupuncture at points such as Hegu (LI4) and Houxi (SI3) activates Aβ fibers to trigger the spinal “gate control” effect,^[15,16]^ combined with 2/100 Hz sparse-dense wave electroacupuncture to promote the release of endorphins/enkephalins,^[17,18]^ forming a dual analgesic network of “peripheral blockade-central regulation,” resulting in a significant reduction in resting/cough VAS scores within 48 hours postoperatively and a 37.2% reduction in sufentanil requirements^[19,20]^; In the prevention and treatment of PONV, acupuncture stimulation of Neiguan (PC6) inhibits the medullary vomiting center and 5-HT3 release,^[21]^ synergizing with the opioid-sparing effect of a 42% reduction in remifentanil dosage,^[22]^ significantly reducing postoperative nausea scores from 4 to 24 hours; In response to surgical stress, acupuncture regulates the hypothalamic-pituitary-adrenal axis to inhibit excessive secretion of CRH/glucocorticoids,^[23]^ combined with ESPB to block sympathetic nerve output, synergistically reducing inflammatory factors such as IL-6 and TNF-α,^[24]^ significantly limiting the increase in neutrophil counts within 24 hours postoperatively (*P* < .01), and maintaining immune homeostasis. Although previous studies have demonstrated that electroacupuncture may reduce the release of inflammatory cytokines and modulate the hypothalamic-pituitary-adrenal axis, the present study did not directly assess biomarkers such as IL-6, TNF-α, or cortisol. Therefore, the mechanistic explanations provided are based on existing literature and should be interpreted as theoretical rather than experimentally verified in this cohort.

Several potential sources of bias inherent to retrospective studies should be acknowledged. First, although all patients underwent thoracoscopic lobectomy, variations in surgical technique and differences among operating surgeons may have influenced postoperative pain and stress responses. Second, subtle differences in double-lumen endotracheal tube positioning or bronchial cuff pressure may affect airway irritation and opioid requirements. Third, intraoperative anesthetic titration, although guided by institutional protocols, may still differ among anesthesia providers and act as a confounding factor. Baseline differences that were not captured in the medical records, such as pain sensitivity, psychological status, or intraoperative sympathetic responses, may also contribute to variability. These factors should be considered when interpreting the comparative outcomes between the 2 groups.

## 5. Limitations and prospects

This study had a small sample size and did not include long-term follow-up on the effects of acupuncture on immune function. Future studies should be conducted as multicenter, large-scale trials to further optimize acupoint combinations and electroacupuncture parameters, and explore the long-term benefits of acupuncture in thoracic surgery.

## 6. Conclusion

Acupuncture anesthesia combined with ESPB is a safe and effective multimodal analgesia regimen for patients undergoing thoracoscopic lobectomy. It significantly reduces postoperative pain and nausea, decreases perioperative opioid use, suppresses surgical stress responses, and shortens postoperative hospital stay. This approach integrates the wisdom of traditional medicine with the advantages of modern regional block techniques, providing new evidence-based options for optimizing anesthesia management in thoracic surgery and accelerating patient recovery.

## Author contributions

**Conceptualization:** Jian Huang, Shibiao Chen, Liqin Wang, Weicheng Liu, Yanping Zhan, Mengying Xiong, Jin Zhang.

**Data curation:** Jian Huang, Shibiao Chen, Liqin Wang, Weicheng Liu, Yanping Zhan, Mengying Xiong, Jin Zhang.

**Formal analysis:** Jian Huang, Shibiao Chen, Liqin Wang, Weicheng Liu, Yanping Zhan, Mengying Xiong, Jin Zhang.

**Funding acquisition:** Jian Huang, Liqin Wang, Weicheng Liu, Yanping Zhan, Jin Zhang.

**Investigation:** Jian Huang, Jin Zhang.

**Writing – original draft:** Jian Huang, Weicheng Liu, Yanping Zhan, Mengying Xiong, Jin Zhang.

**Writing – review & editing:** Jian Huang, Weicheng Liu, Yanping Zhan, Mengying Xiong, Jin Zhang.
